# Supplementary material for: Characterizing emergency clinician engagement with social drivers of health data among patients with opioid use disorder
Source: J Am Med Inform Assoc. 2026 Jun 17;33(9):1693–701. doi: 10.1093/jamia/ocag093 (PMC13580726; doi:10.1093/jamia/ocag093)
Supplement: Supplementary file 1 [file ocag093_supplementary_data.docx]

**Appendix A. ICD-10 Social Risk Z Codes and their Social Risk Categorization**

| Z Code | Social Risk Category |
| --- | --- |
| Z59.5 | Finances |
| Z59.6 | Finances |
| Z59.7 | Finances |
| Z59.9 | Finances |
| E63.9 | Food |
| Z59.4 | Food |
| Z59.41 | Food |
| Z73.8 | Food |
| Z73.89 | Food |
| Z59.0 | Housing |
| Z59.00 | Housing |
| Z59.01 | Housing |
| Z59.02 | Housing |
| Z59.1 | Housing |
| Z59.11 | Housing |
| Z59.19 | Housing |
| Z59.8 | Housing |
| Z59.811 | Housing |
| Z59.812 | Housing |
| Z59.819 | Housing |
| Z59.82 | Housing |
| Z59.86 | Housing |
| Z59.861 | Housing |
| Z59.868 | Housing |
| Z59.869 | Housing |
| Z59.87 | Housing |
| Z59.89 | Housing |
| Z77.0 | Housing |
| Z77.011 | Housing |
| Z77.1 | Housing |
| Z77.120 | Housing |
| O9A.3 | Safety |
| O9A.31 | Safety |
| O9A.311 | Safety |
| O9A.312 | Safety |
| O9A.313 | Safety |
| O9A.319 | Safety |
| O9A.32 | Safety |
| O9A.33 | Safety |
| O9A.4 | Safety |
| O9A.41 | Safety |
| O9A.411 | Safety |
| O9A.412 | Safety |
| O9A.413 | Safety |
| O9A.419 | Safety |
| O9A.42 | Safety |
| O9A.43 | Safety |
| O9A.5 | Safety |
| O9A.51 | Safety |
| O9A.511 | Safety |
| O9A.512 | Safety |
| O9A.513 | Safety |
| O9A.519 | Safety |
| O9A.52 | Safety |
| O9A.53 | Safety |
| R45.8 | Safety |
| R45.82 | Safety |
| S09.9 | Safety |
| S09.90 | Safety |
| T14.9 | Safety |
| T14.90 | Safety |
| T74.0 | Safety |
| T74.02 | Safety |
| T74.1 | Safety |
| T74.11 | Safety |
| T74.12 | Safety |
| T74.2 | Safety |
| T74.21 | Safety |
| T74.22 | Safety |
| T74.3 | Safety |
| T74.31 | Safety |
| T74.32 | Safety |
| T74.9 | Safety |
| T74.91 | Safety |
| T74.92 | Safety |
| T76.0 | Safety |
| T76.02 | Safety |
| T76.1 | Safety |
| T76.11 | Safety |
| T76.12 | Safety |
| T76.2 | Safety |
| T76.21 | Safety |
| T76.22 | Safety |
| T76.3 | Safety |
| T76.31 | Safety |
| T76.32 | Safety |
| T76.91 | Safety |
| X58 | Safety |
| Z60.8 | Safety |
| Z62.8 | Safety |
| Z62.81 | Safety |
| Z62.9 | Safety |
| Z63.0 | Safety |
| Z65.4 | Safety |
| Z74.8 | Transportation |

**Appendix B. Encounters Meeting Opioid Use Disorder Phenotype Criteria, by Criterion**

|  | Count | Proportion^a^ |
| --- | --- | --- |
| ≥1 ICD-10 codes indicating OUD, withdrawal, or overdose in encounter (discharge) diagnoses, problem lists, or past medical history | 3228 | 0.566 |
| Positive urine toxicology results for methadone, fentanyl, or heroin | 1987 | 0.349 |
| ED clinician notes (including triage nursing notes) containing “OUD”, “opioid use disorder”, “opioid use d/o”, “COWS”, “fentanyl”, and/or “heroin” | 1701 | 0.298 |
| Active prescriptions for buprenorphine or methadone preceding the index ED visit encounter | 955 | 0.168 |
| Addiction care team consultation notes with reason for consult containing “heroin”, “opioid”, “opiate”, “Narcan”, and/or “fentanyl” | 562 | 0.099 |
| Reason for visit free text containing “heroin”, “opioid”, “opiate”, and/or “OUD”, “fentanyl” (excluding use together with phrases suggesting therapeutic use or EMS-only administration), “fentanyl use” | 493 | 0.086 |
| ED admission diagnoses containing “heroin”, “opioid”, “opiate”, and/or “OUD”, “fentanyl” (excluding use together with phrases suggesting therapeutic treatment or EMS-only administration), “fentanyl use” and/or mapping to an ICD-10 code for OUD | 36 | 0.006 |

**Abbreviations:** ICD-10, International Classification of Diseases, Tenth Revision; OUD, opioid use disorder; COWS, Clinical Opiate Withdrawal Scale; EMS, emergency medical services; ED, emergency department

^a^Proportions do not add to 1 due to encounters meeting more than one phenotype criteria
